# F84. ASSOCIATIONS BETWEEN INTELLIGENCE, VERBAL WORKING MEMORY AND PROCESSING SPEED IN PARENTS WITH SCHIZOPHRENIA OR BIPOLAR DISORDER AND THEIR 7-YEAR OLD OFFSPRING

**DOI:** 10.1093/schbul/sby017.615

**Published:** 2018-04-01

**Authors:** Aja Greve, Jens Richardt Møllegaard Jepsen, Erik Lykke Mortensen, Rudolf Uher, Lynn Mackenzie, Leslie Foldager, Ditte Gantriis, Birgitte Klee Burton, Ditte Ellersgaard, Camilla Jerlang Christiani, Katrine Spang, Nicoline Hemager, Maria Toft Henriksen, Kate Kold Zahle, Henriette Stadsgaard, Anne Thorup, Merete Nordentoft, Kerstin Plessen, Ole Mors, Vibeke Bliksted

**Affiliations:** 1 Aarhus University Hospital; 2 Copenhagen University Hospital; 3 University of Copenhagen; 4 Dalhousie University, Halifax; 5 Aarhus University; 6 Child and Adolescent Mental Health Centre, Mental Health Services Capital Region; 7 Mental Health Centre Copenhagen, Copenhagen University Hospital, Mental Health Services Capital Region

## Abstract

**Background:**

Neurocognitive phenotypes may contribute to understanding the pathway leading from genes to psychopathology. We aimed to investigate associations of intelligence, processing speed and verbal working memory between parents and offspring in families with schizophrenia, bipolar disorder and controls.

**Methods:**

Data are from The Danish High Risk and Resilience Study – VIA7, a population-based nationwide cohort identified through Danish Registries. Participants are 522 children aged 7 with no, one, or two parents with schizophrenia or bipolar disorder and biological parents. Control children were matched to children from the schizophrenia group (age, gender, and municipality). Children at familial risk of bipolar disorder were comparison group. Child assessors were blinded to risk status. Main Outcomes were intelligence measured with Reynolds Intellectual Screening Test (RIST), verbal working memory assessed with letter number sequencing (LNS) and processing speed assessed with Coding (WISC-IV/WAIS-IV).

**Results:**

We examined 434 index parents (151 schizophrenia, 100 bipolar disorder and 183 controls, mean (SD) age 39.7 (5.7), 264 (61%) females), 443 co-parents (mean (SD) age 40.1 (5.4), 210 (47%) females) and 489 children (mean (SD) age 7.8 (0.2), 231 (47%) females). Children′s intelligence was associated with index parents’ intelligence (B = 0.40, 95% CI: 0.28;0.52, p < 0.001) and co-parents’ intelligence (B = 0.16, 95% CI: 0.03;0.28, p = 0.012). Children’s processing speed was associated with index parents’ processing speed (B = 0.07, 95% CI: 0.02;0.12, p = 0.007), co-parents’ processing speed (B = 0.09, 95% CI: 0.04;0.15, p < 0.001), group (schizophrenia: B = -1.92, 95% CI: -3.63;-0.21, p = 0.028) and gender of child (male: B = -4.55, 95% CI: -4.98; -2.12, p < 0.001). Children’s working memory was associated with index parents’ LNS score (B=0.25, 95% CI: 0.13;0.37, p < 0.001), co-parents’ working memory (B = 0.23, 95% CI: 0.09;0.37, p = 0.001), group (schizophrenia: B = -1.02, 95% CI: -0.89;-0.16, p = 0.020) and gender of child (male: B = -0.85, 95% CI: -1.60;-0.11, p = 0.025).

**Discussion:**

Findings showed associations of neurocognitive phenotypes between parents and offspring. These associations do not differ markedly between schizophrenia, bipolar disorder and controls.

